# Case report: Pulmonary syphilis mimicking pulmonary hematogenous metastases on chest CT and integrated PET/CT

**DOI:** 10.4103/0971-3026.76052

**Published:** 2011

**Authors:** Hyung Jun Kim, Hyun Ju Seon, Hyo Hyun Shin, Yoo-Duk Choi

**Affiliations:** Department of Radiology, Chonnam National University Hospital, Gwangju, South Korea; 1 Department of Pathology, Chonnam National University Hospital, Gwangju, South Korea

**Keywords:** Chest CT, ^18^F FDG PET-CT, pulmonary syphilis

## Abstract

We report a case of syphilis with pulmonary involvement. Chest CT scan and ^18^F-fluorodeoxyglucose (FDG) PET/CT showed multiple pulmonary nodules mimicking pulmonary hematogenous metastases. This was confirmed on follow-up images that showed therapeutic response to penicillin.

## Introduction

Syphilis is a sexually transmitted disease caused by a spirochete named *Treponema pallidum*, which can damage the heart, aorta, brain, eyes, and bones when inadequately treated.[1–3] A few cases of pulmonary syphilis have been reported with varying imaging findings.[4–6] We would like to describe a case of pulmonary syphilis mimicking pulmonary hematogenous metastases on initial noninvasive diagnostic work-up.

## Case Report

A 59-year-old woman visited our hospital, suffering from right upper quadrant abdominal pain for 9 h. The patient also complained of 4 kg weight loss during the last month and a 1-week history of cough and sputum. Physical examination showed tenderness and rebound tenderness in the right upper quadrant of the abdomen and palpable lymphadenopathy in both inguinal areas. There was no demonstrable skin rash or genital ulcer.

**Figure 1 (A-C):**
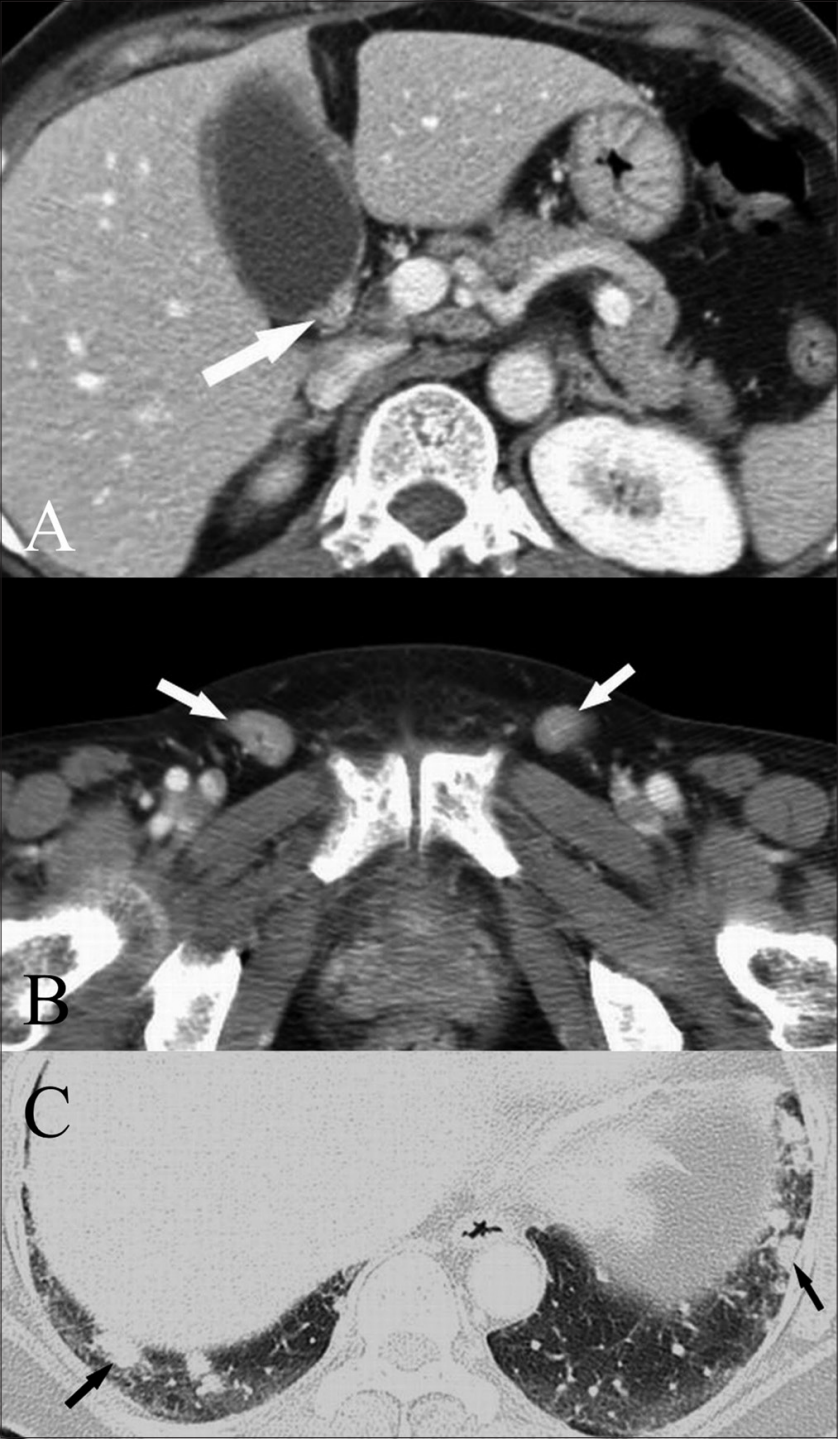
Contrast-enhanced CT scan of the abdomen (A) and pelvis (B) shows irregular wall thickening in the neck of the gallbladder (arrow in A), with multiple, enlarged inguinal lymph nodes (arrows in B) showing relatively preserved fatty hila. CT scan of the chest (C) shows multiple, well-defined, small, subcentimetre nodules (arrows), in both lower lobes

An initial contrast-enhanced abdominal CT scan showed several gallbladder stones and diffuse wall thickening of the gallbladder, especially with irregular thickening in its neck [Figure 1A], suggesting possible malignancy. Several enlarged lymph nodes with relatively preserved fatty hilum were seen in both inguinal regions [Figure 1B], along both iliac vessels, and in the portocaval space (not shown). Multiple pulmonary nodules were noted as well. A subsequent chest CT scan revealed multiple, small, well-defined nodules (<1 cm in diameter), in the right upper lobe (not shown) and both lower lobes [Figure 1C], suggesting possible metastatic disease.

**Figure 2 (A,B):**
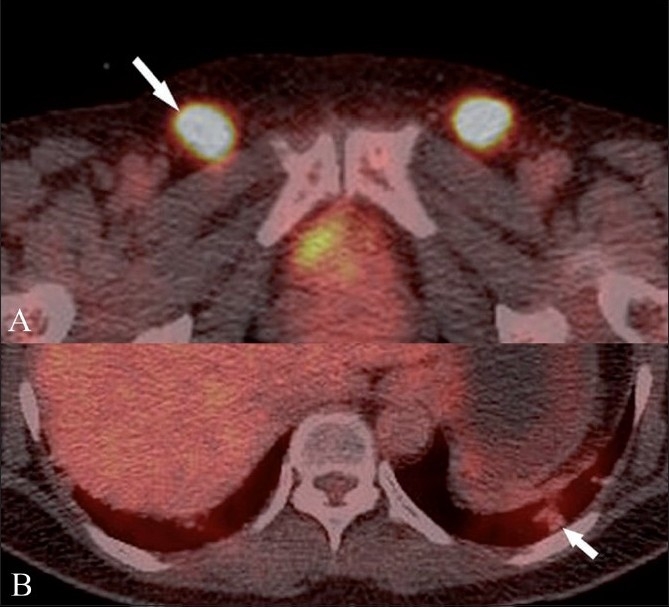
FDG PET/CT shows increased uptake in the inguinal lymph nodes (arrow in A) with no significant uptake in the lung nodules (arrow in B)

An ^18^F FDG PET/CT scan showed hypermetabolic enlarged nodes (standardized uptake value – SUV of 10) in both inguinal regions [Figure 2A], along both iliac vessels and the portacaval space (not shown). However, there was no significant hypermetabolism (maximum SUV: 1.2) in the small pulmonary nodules [Figure 2B].

A preoperative laboratory screening showed the rapid plasma reagin (RPR) and venereal disease research laboratory (VDRL) tests to be positive. These were followed by more specific treponemal tests; the *Treponema pallidum* hemagglutination assay (TPHA) (titer, 1:302) was positive and the fluorescent Treponemal antibody absorption (FTA-ABS) test was negative for IgM and positive for IgG. Therefore, a clinical diagnosis of syphilis was made.

There was no evidence of other infection or malignancy in the preoperative image studies, physical examination, and laboratory findings, including sputum examination.

**Figure 3:**
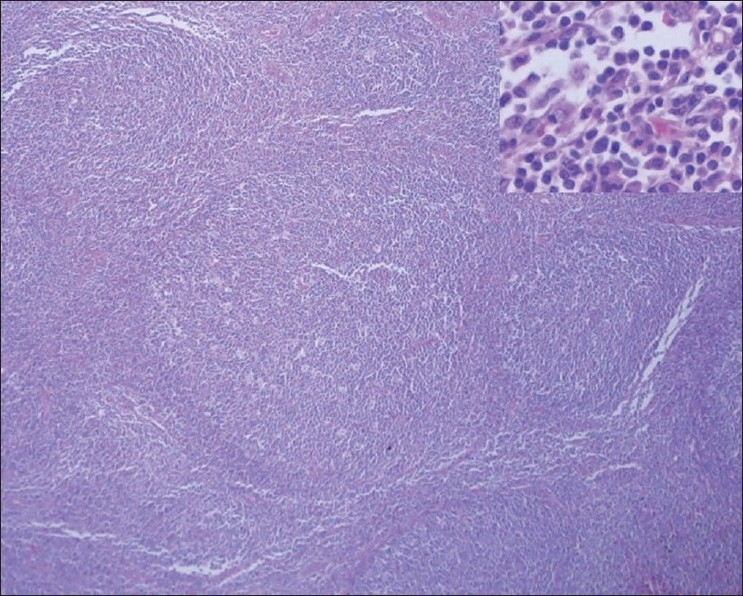
Histopathology of the inguinal lymph node shows marked hyperplasia of secondary lymphoid follicles and extensive plasma cell proliferation (inset) in the interfollicular areas (H and E, ×20)

Cholecystectomy and left inguinal lymph node biopsy were performed. The histologic examination of the gallbladder confirmed chronic cholecystitis. The histologic examination of a left inguinal lymph node specimen showed a negative Warthin Starry silver stain, but revealed hyperplasia of secondary lymphoid follicles and extensive plasma cell proliferation on hematoxylin-eosin stain, suggesting possible syphilis [Figure 3].

**Figure 4 (A,B):**
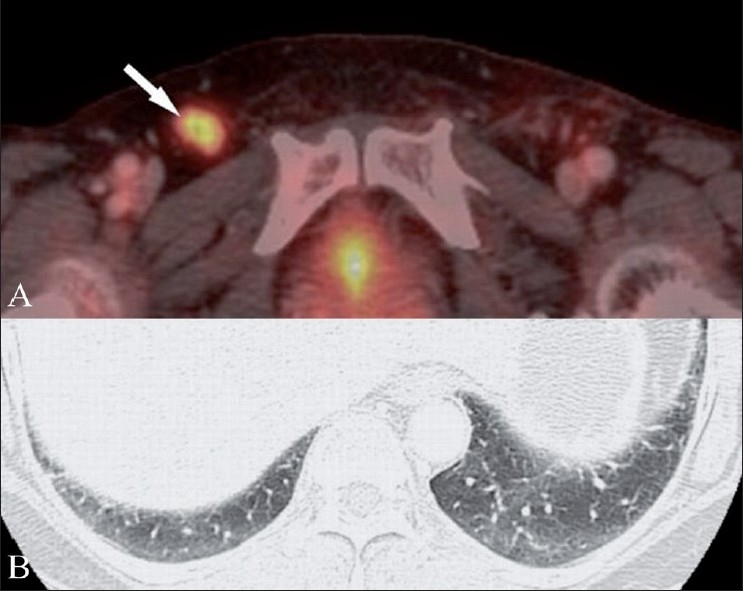
Follow-up FDG PET/CT (A) after penicillin therapy shows decreased FDG uptake in the right inguinal lymph node (arrow). Follow-up CT scan of the chest (B) obtained 2 months after discharge shows complete disappearance of the pulmonary nodules

Treatment with intramuscular (IM) penicillin G was initiated. After 1 week, PET/CT scan showed a decrease in the FDG uptake (max SUV - 5.2) in the involved lymph nodes [Figure 4A]. Two months after discharge, the patient returned for a follow-up evaluation and a CT scan showed complete disappearance of the pulmonary nodules [Figure 4B] with a further reduction in the size of the involved lymph nodes.

## Discussion

Pulmonary syphilis is very rare. In the preantibiotic period, the prevalence of lung involvement mainly in congenital and tertiary syphilis varied from 1% to 12.5%.[4,7–9] However, the prevalence of secondary syphilis has increased substantially during the past years.[10] Mucocutaneous manifestations are easily recognized, and occur in 80% of cases. Less commonly, secondary syphilis may present with pulmonary infiltration, acute meningitis, sensorineural hearing loss, iritis, gastropathy, nephritic syndrome, and periostitis.[1] Since 1967, there have been 10 case reports of pulmonary involvement in secondary syphilis.[9] The clinical criteria proposed by Coleman *et al*.[4]

For diagnosing pulmonary syphilis are:

1. historical and physical findings typical of syphilis,
2. serologic test results positive for syphilis,
3. pulmonary involvement seen radiologically with or without associated symptoms or signs,
4. exclusion of other forms of pulmonary disease, when possible, according to findings of serological tests, sputum smears and cultures, and cytological examination of sputum, and
5. response to penicillin of signs found on radiological examination. Clinical and radiological response to penicillin remains the best confirmation of pulmonary syphilis and can be of help in narrowing the differential diagnosis.[9]

Radiological presentation described in the English literature includes solitary and multiple pulmonary nodules and infiltrates, occasionally associated with pleural effusion,[9] findings that may also be seen in other benign or malignant conditions such as metastases, lymphoma, Kaposi sarcoma, Wegener granulomatosis, sarcoidosis, rheumatoid arthritis, tuberculosis, invasive aspergillosis, histoplasmosis, coccidiomycosis, and septic emboli. To confirm the diagnosis, serologic and biologic tests, bronchoalveolar lavage, and lung biopsy may be useful. Our patient presented with multiple subcentimetre pulmonary nodules, which in the setting of presumed gall-bladder malignancy, suggested possible metastatic disease. The diagnosis was clinched by the virtually complete resolution of the nodules on a follow-up chest CT scan, after adequate penicillin therapy.

FDG-PET assesses increased glucose metabolism in lesions and may show uptake in a large variety of primary lung tumors, metastases as well as in inflammatory diseases such as tuberculosis, fungal infection and sarcoidosis.[11] Although the overall sensitivity and specificity of FDG-PET in pulmonary lesions are high, it has a lower accuracy in smaller, subcentimetre lung lesions.[11,12] In our patient, there was no uptake in the small nodules, probably due to their small size.

FDG can also accumulate in pelvic lymph nodes affected by inflammatory and neoplastic conditions[13,14] and may not be able to differentiate between them.[15] Our patient had increased FDG uptake in pelvic nodes with subsequent therapeutic response to penicillin.

In conclusion, given that pulmonary syphilis is rarely encountered, we have demonstrated its appearance on CT scan and PET/CT in a patient where metastatic disease was the initial presumed diagnosis.
